# Eomesodermin in CD4^+^T cells is essential for Ginkgolide K ameliorating disease progression in experimental autoimmune encephalomyelitis

**DOI:** 10.7150/ijbs.50041

**Published:** 2021-01-01

**Authors:** Sheng Chen, Juan Zhang, Wen-Bo Yu, Jing-Cong Zhuang, Wei Xiao, Zhi-Ying Wu, Bao-Guo Xiao

**Affiliations:** 1 Department of Neurology, Huashan Hospital, Fudan University.; 2 Department of Neurology and Research Center of Neurology, Second Affiliated Hospital, Key Laboratory of Medical Neurobiology of Zhejiang Province, Zhejiang University School of Medicine.; 3 Department of Neurology, Fujian Medical University Union Hospital.; 4 Department of Neurology and Institute of Neurology, First Affiliated Hospital, Fujian Medical University.; 5 Key Laboratory of New-tech for Chinese Medicine Pharmaceutical Process, Lianyungang, China.

**Keywords:** Multiple sclerosis· Transcription factors· EAE· Therapeutic target

## Abstract

Eomesodermin (Eomes), a transcription factor, could suppress the Th17 cell differentiation and proliferation through directly binding to the promoter zone of the *Rorc* and* Il17a* gene, meanwhile the expression of *Eomes* is suppressed when c-Jun directly binds to its promoter zone. Ginkgolide K (1,10-dihydroxy-3,14-didehydroginkgolide, GK) is a diterpene lactone isolated from the leaves of Ginkgo biloba. A previous study indicated that GK could decrease the level of phospho JNK (c-Jun N-terminal kinase). Here, we reported the therapeutic potential of Ginkgolide K (GK) treatment to ameliorate experimental autoimmune encephalomyelitis (EAE) disease progression.

**Methods:** EAE was induced in both wildtype and CD4-*Eomes* conditional knockout mice. GK was injected intraperitoneally. Disease severity, inflammation, and tissue damage were assessed by clinical evaluation, flow cytometry of mononuclear cells (MNCs), and histopathological evaluation. Dual-luciferase reporter assays were performed to measure Eomes transcription activity in vitro. The potency of GK (IC_50_) was determined using JNK1 Kinase Enzyme System.

**Results:** We revealed that GK could ameliorate EAE disease progression by the inhibition of the Th17 cells. Further mechanism studies demonstrated that the level of phospho JNK was decreased and the level of Eomes in CD4^+^T cells was dramatically increased. This therapeutic effect of GK was almost completely interrupted in CD4-*Eomes* conditional knockout mice.

**Conclusions:** These results provided the therapeutic potential of GK treatment in EAE, and further suggested that Eomes expression in CD4^+^T cells might be essential in this process.

## Introduction

Multiple sclerosis (MS) is a central nervous system (CNS) autoimmune disorder characterized by chronic demyelinating of brain and spinal cord 1. Experimental autoimmune encephalomyelitis (EAE) is the commonly used mice model for MS. The demyelination of EAE is mainly mediated by T helper 1 (Th1) and T helper 17 (Th17) cells. A novel therapy for suppressing the Th1 or Th17 cells could be beneficial for the treatment of EAE, as well as MS 2.

As indicated previously, several transcription factors (TFs) are playing critical roles in the differentiation of Th cells. T-bet is a major factor for Th1 cell differentiation and IFN-γ production^3^. Similarly, Foxp3 is the master transcription factors of the regulatory T (Treg) cell 4, 5. Th17 cells, characterized by the production of IL-17A, IL-17F, and IL-21 6, 7, do not express T-bet or Foxp3, instead they express high levels of RORγt 6, 8. Nearly 50% of activated RORγt overexpression cells produce IL-17. Furthermore RORγt-deficient mice are resistant to EAE 9. The RORγt is mainly induced by TGF-β, together with IL-6, via the SMAD pathway 8, 9. Nevertheless, Ichiyama and colleagues have already demonstrated that Eomesodermin (Eomes), a transcription factor, could directly bind to the promoter zone of *Rorc* and *Il17a* gene, which suppresses the Th17 cell differentiation and proliferation. In this Smad-independent pathway, the expression of *Eomes* gene is dramatically suppressed when c-Jun directly binds to its promoter zone 10.

Ginkgolide K (1,10-dihydroxy-3,14-didehydroginkgolide, GK) is a diterpene lactone isolated from the leaves of *Ginkgo biloba*. Our previous study revealed that GK treatment could ameliorate EAE progression by inhibiting the infiltration of inflammatory cells and demyelination in the spinal cord 11. In the same study, we found GK could inhibit the proliferation of reactive CD4^+^T cells by CFSE labeling and decrease the proportion of CD4^+^IL-17^+^T cells ex vivo 11. However, the underlying mechanism remained unconcluded. Previous study indicated that GK, as a low molecular weight (MW) compound, could decrease the level of phospho JNK (c-Jun N-terminal kinase) in neonatal rat cardiomyocytes (NRMCs) 12. Since c-Jun is one of the major downstream TFs of phospho-JNK (c-Jun N-terminal kinase), we assumed that GK treatment may increase the expression of *Eomes* and inhibit the development of Th17 cells in EAE according to the previous report 10, resulting in an amelioration of EAE disease progression. Furthermore, we hope to clarify whether the expression of* Eomes* is essential in the process of GK action during EAE development. To identify these questions, we firstly tested whether GK could ameliorate EAE disease progression in the wild-type and *Eomes* conditional ablation (CD4-*Eomes*^fl/fl^) EAE model, then explored the role of Eomes playing in this process. The results showed that the disease progression was significantly ameliorated in wildtype mice with elevated expression of *Eomes*. Interestingly, Eomes deficiency in CD4^+^T cells restricted the effect of GK treatment.

## Materials and Methods

### Animals

C57BL/6 *CD4-Cre* (Stock No: 022071) and *Eomes* ^fl/fl^ (Stock No: 017293) mice were introduced from *The Jackson Laboratory* (www.jax.org) and maintained under specific pathogen-free conditions at *Shanghai Research Center for Model Organisms* (http://www.biomodel.com.cn) in accordance with institutional guidelines. The study was approved by the Ethics Committee of Fudan University Ethics Committee (Approval No. 20171542A493).

### EAE induction, clinical evaluation and GK treatment

For EAE induction, female mice (10-12 weeks old) were immunized subcutaneously with 300μg MOG_35-55_ (Chinapeptides, China) emulsified in complete Freund's adjuvant (CFA) (100μg/mouse, supplemented with 5mg/ml heat-inactive Mycobacterium tuberculosis H37RA) and injected intraperitoneally with 300ng pertussis toxin (List Biological Laboratories, USA) at the same time of immunization and again 48h after immunization. EAE were daily assessed for clinical signs of disease (0, no signs of EAE; 1, complete flaccid tail; 2, flaccid tail and hind limb weakness; 3, severe hind limb weakness; 4, bilateral hind limb paralysis; 5, complete hind limb paralysis and forelimb weakness; 5.5, paralysis of both fore limbs and hind limbs; 6, moribund state or death). Ginkgolide K (GK, CAS NO. 153355-70-5) was provided by Key Laboratory of New-tech for Chinese Medicine Pharmaceutical Process, Lianyungang, China. For GK preparation, 15mg GK powder was dissolved in 3.5ml PEG400 by ultrasonic disrupter and 0.5ml ethanol was subsequently added. Other 6ml saline was added to make a total volume to 10ml before use. For treatment, GK (15mg/kg bodyweight) was injected intraperitoneally once per day from day 3 to day 26 post-immunization (p.i.). The control group was injected intraperitoneally with vehicle (35% PEG400+5% ethanol+60% saline) in the same manner.

### Immunohistochemistry and inflammation evaluation

EAE mice were anesthetized with 1.5% Isoflurane before sacrificed. The spinal cords were dissected and fixed in 4% paraformaldehyde overnight and O.C.T. compound embedded. Sections were stained using H&E and Luxol fast blue. The evaluation of histological inflammation (inflammatory index) was assessed as described previously 13, 14. The detail inflammatory index is as follows: 0, no inflammation in the CNS;1, a few infiltrated inflammatory cells in the perivascular areas and meninges; 2, mild cellular infiltration in the parenchyma; 3, moderate cellular infiltration in the parenchyma; 4, severe cellular infiltration in the parenchyma. The histological inflammation was evaluated by three experienced examiners using three different sections respectively.

### Cell isolation and flow cytometry

At day 9, 18 and 28 p.i., cell suspensions from spleen and lymph node were prepared respectively by mechanical disruption of tissues and isolated using Mouse Lymphocyte Separation Medium (DAKEWE, China). Mononuclear cells (MNCs) were cultured in RPMI-1640 medium and stimulated with 10μg/ml MOG_35-55_ for 48 hours. For intracellular IFN-γ, IL-17A and Eomes staining, cells were re-stimulated with eBioscience Cell Stimulation Cocktail (eBioscience, USA) for additional 5 hours. Cells were fixed and permeabilized using eBioscience Foxp3 staining kit (eBioscience, USA), and stained with anti-CD4 Ab (RM4-5; eBioscience), anti-IFN-γ Ab (XMG1.2; eBioscience), anti-IL-17A Ab (TC11-18H10.1, BioLegend) and anti-Eomes Ab (Dan11mag; eBioscience) according to the manufacturer's instructions. For flow cytometric analysis of JNK1/2 (pT183/pY185,), mice splenocytes were re-stimulated with PMA (Sigma, 400 nM), and Ionomycin (Sigma, 250 ng/ml) at 37˚C for 15 minutes according to the manufacturer's instructions and previous study 15. Then cells were fixed in 1× BD Phosflow™ Lyse/Fix Buffer (10 minutes at 37˚C) and permeabilized in BD Phosflow™ Perm Buffer III on ice for 30 minutes. Cells were then stained with anti-JNK (pT183/pY185) ab (562481, BD Bioscience). Appropriate fluorescein-conjugated, isotype-matched, irrelevant mAbs were used as negative controls. Cells were acquired on Attune NxT Flow Cytometer (Thermo Fisher Scientific, USA) and analyzed by FlowJo VX Software (FlowJo, LLC).

### Cytokine ELISA assay

The concentration of cytokines in serum of mice was measured by IL-17A (PeproTech), IFN-γ (PeproTech) and IL-6 (eBioscience) ELISA kits following the manufacturer's protocol respectively.

### Cell line and cell culture

The HEK293T cells for Luciferase assays were cultured in DMEM, supplemented with 10% FBS (Gibco, USA), 100U/ml penicillin, and 100mg/ml streptomycin at 37˚C in a humidified atmosphere containing 5% CO_2_.

### Plasmid construction and Luciferase assays

The cDNA encoding human c-Jun was prepared using PCR fragments from human cDNA and subcloned into pFLAG-CMV-4 vector. Luciferase reporter genes containing human *TBX21*, *RORC* and *EOMES* promoter sequences were prepared using PCR fragments from human genomic DNA. The PCR products were subsequently subcloned into the pGL4.27 vector (Promega). The HEK293T cells were seeded into 24-well plates in advance and transfected with the reporter plasmid and pRL-TK plasmid using Lipofectamine 3000 reagent (Invitrogen). After 24 hours, the cells were lysed and analyzed using a dual luciferase reporter assay (E2910, Promega) according to the manufacturer's instructions.

### Prediction of GK protein docking and JNK kinase assay

To predict whether the low MV compound GK could directly bind to a specific protein, an online prediction and analysis was performed using systemsDock 16. For JNK kinase assay, GK was dissolved in 5% DMSO. GK dose response was created according to the manufacturer's instructions using JNK1 Kinase Enzyme System plus ADP-Glo™ (V4071, Promega) to determine the potency of GK (IC_50_).

### Quantification real-time PCR (qRT-PCR) analysis

For qRT-PCR analysis, total RNAs were extracted from cells using RNAiso Plus (Takara, Japan) according to the manufacturer's instructions. The cDNAs were synthesized using the PrimeScript™ RT Master Mix Kit (Takara, Japan). The mRNA expression was measured using Roche Cobas Z480 system (Roche, Switzerland) with our in-house designed primers (Supplemental Table 1). The relative expression was determined as ΔCT, and the fold change in gene expression was calculated with the 2^-ΔΔCT^ method.

### Western blot analysis

Protein extracts (30μg) from splenocytes were separated by SDS-PAGE, and transferred onto a Nitrocellulose Blotting Membrane (GE healthcare Life Science, Germany). Then the membranes were incubated with rabbit anti-phospho c-Jun (2361, Cell Signaling Technology), rabbit anti-c-Jun (9165, Cell Signaling Technology) and rabbit anti-lamin B (Abmart). Bands were visualized by HRP conjugated secondary antibodies and chemiluminescence (ECL) kit under ECL system (Bio-Rad Laboratories).

### Statistical analyses

Statistical analyses were performed with GraphPad Prism 8.01. Statistical significance was measured using nonparametric Mann-Whitney test. Differences were considered statistically significant when p<0.05.

## Results

### GK ameliorates the disease progression of EAE

**Figure 1:**
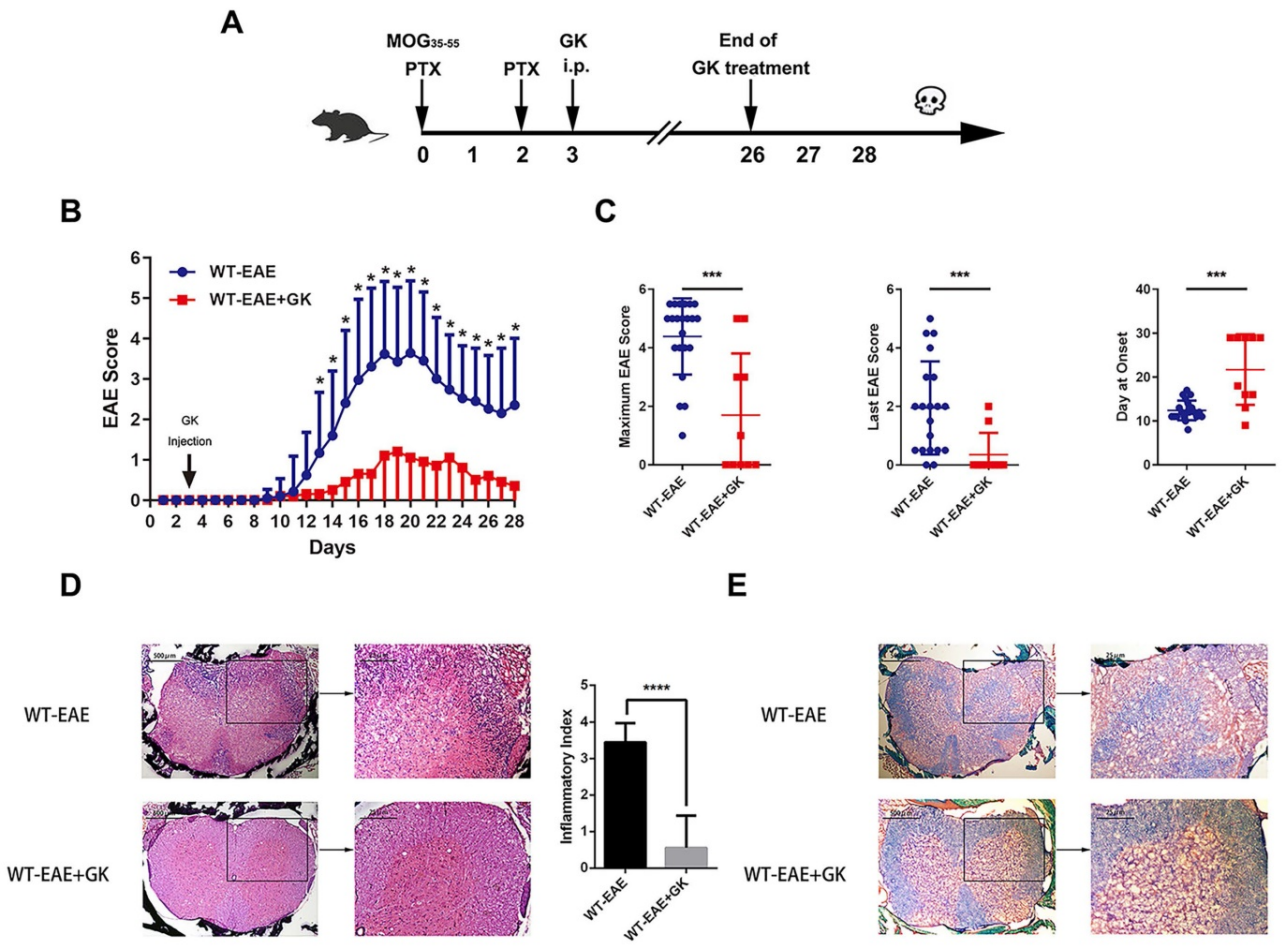
** Ginkgolide K (GK) treatment ameliorates the disease progression of EAE.** (A) Wildtype C57BL/6 mice were immunized with MOG_35-55_ and injected intraperitoneally with 15mg per kilogram bodyweight GK once per day from day 3 p.i. to day 26 p.i. (B) EAE clinical score of GK treatment group (n=10) and vehicle control group (n=20). (C) The maximum EAE score, last EAE score and average day at onset of GK treatment group and vehicle control group. (D) H&E staining of GK treatment group and vehicle control group. The histological inflammation index was evaluated using three different sections. (E) Luxol fast blue plus eosin staining of GK treatment group and vehicle control group. Data are representative of at least three independent experiments. Mean±SD is shown. * p<0.05, ** p<0.01, *** p<0.001, **** p<0.0001.

We performed EAE induction in wildtype mice at first. As was shown in Figure 1A, GK treatment began at day 3 p.i., and ended at day 26 p.i. The results indicated that GK treatment significantly reduced EAE score started at day13 till the end, as compared to vehicle control mice (Figure 1B). As well, the maximum EAE score, the last EAE score and the average day at the disease onset differed significantly between two groups (Figure 1C). Histopathological observation clearly revealed the reduction of inflammation (Figure 1D) and demyelination (Figure 1E) in the affected spinal cord in wildtype EAE mice with GK treatment.

**Figure 2:**
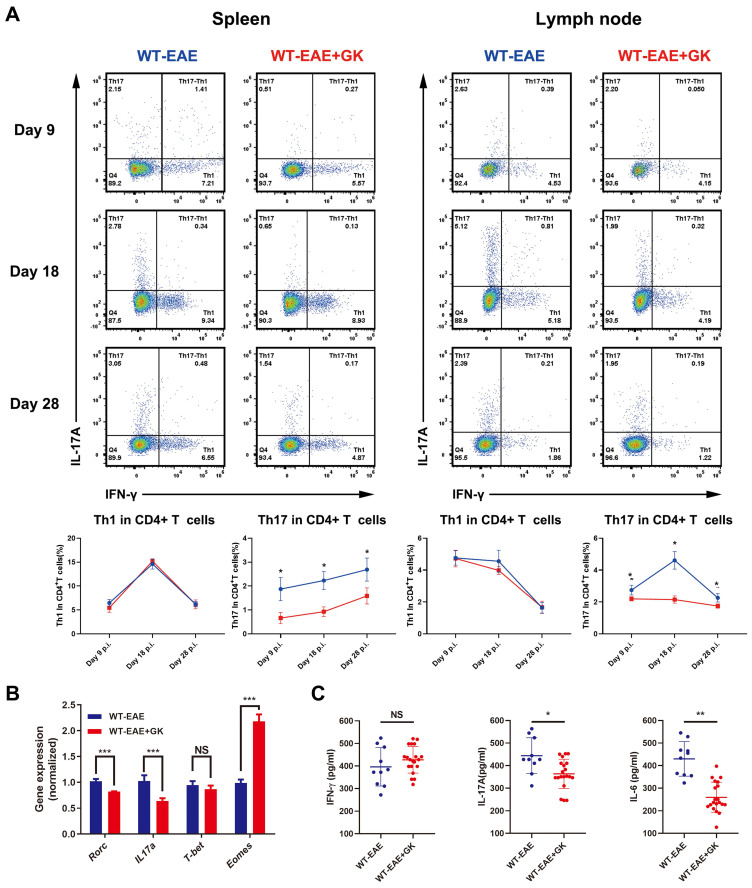
** GK treatment decreased the level of inflammation in EAE.** (A) Intracellular cytokines IL-17A and IFN-γ staining by FACS at day 9 p.i., day 18 p.i. and day 28 p.i. (B) Spleen MNCs gene expression of* Rorc*, *IL17a*, *T-bet* and *Eomes* by qRT-PCR at day 28 p.i. (C) IFN-γ, IL-17A and IL-6 production in the serum of GK treatment group and vehicle control group. Data are representative of at least three independent experiments. Mean±SD is shown. NS Not significant, * p<0.05, ** p<0.01, *** p<0.001.

The intracellular cytokine staining revealed a significant decrease in Th17 cells, instead of Th1 cells, among CD4^+^T cells in both spleen and lymph node at day 9 p.i., 18 p.i. and 28 p.i. (Figure 2A). Additionally, qRT-PCR results indicated that the expression of *Rorc*, the master transcription factor of Th17 cells, as well as the expression of *Il17a* were decreased after GK treatment (Figure 2B). Interestingly, the expression of *Eomes*, instead of *T-bet*, was dramatically increased by more than 2-fold after GK treatment (Figure 2B). As well, ELISA assay revealed a significant decrease in IL-17A and IL-6 production in serum of EAE mice with GK treatment. These results conjointly demonstrated that GK treatment could attenuate the severity of EAE by inhibiting the development of Th17 cells, along with a dramatically change of the *Eomes* gene expression.

### GK could directly binds to JNK and inhibit its kinase activity

**Figure 3:**
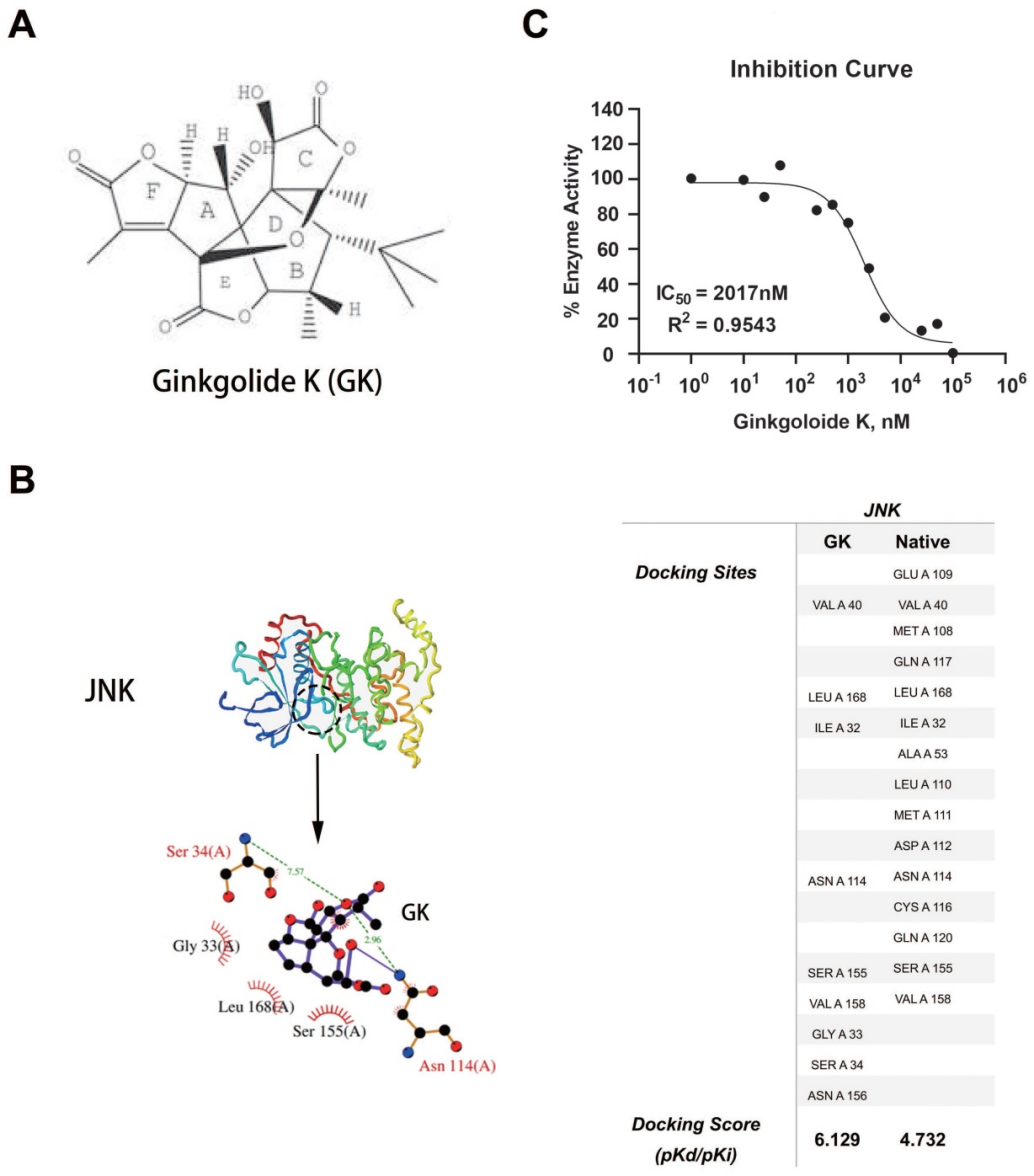
** GK binding simulation and dose response curve.** (A) Molecular structure of GK. (B) 3- dimensional structure of JNK, as well as the prediction docking sites. (C)GK dose response curve with GK in vitro.

Using systemsDock, we performed GK docking simulation with JNK 12. Interestingly, in silico simulations indicated that GK (molecular structure was shown in Figure 3A) might directly bind to JNK (Figure 3B). To test the simulation, we further performed JNK1 kinase assay to determine the inhibition potency of GK. As was shown in Figure 3C, GK could directly inhibit JNK1 kinase activity in vitro, the IC_50_ of which was measured as 2017nM.

### GK increases the expression of *Eomes* through inhibition of phospho JNK

**Figure 4:**
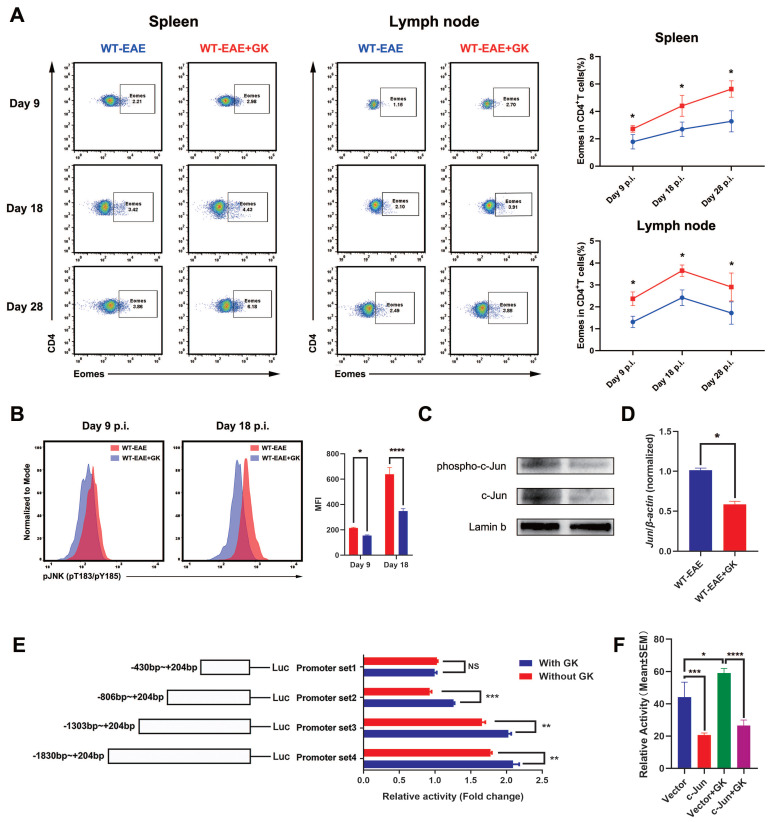
** GK increases the expression of *Eomes* through inhibition of phospho JNK.** (A)Spleen and lymph node CD4^+^ Eomes^+^ T cells of GK treatment group (n=6) and vehicle control group (n=6) at EAE day 9 p.i., day 18 p.i. and day 28 p.i. by FACS. (B) The phospho JNK in spleen CD4^+^T cells at EAE day 9 p.i.and day 18 p.i. (C) The western blot of c-Jun and phospho c-Jun at EAE day 18 p.i. with or without GK treatment. (D) Spleen MNCs *Jun* expression between EAE with or without GK treatment. (E) Different length *hEOMES* promoter luciferase plasmids construction and its transcriptional activities with or without 50μg GK in HEK293T cells. (F) Transcriptional activities of* hEOMES* promoter luciferase plasmid cotransfected with pFlag-c-Jun plasmid in HEK293T cells. Data are representative of at least three independent experiments. Mean±SD is shown. NS Not significant, * p<0.05, ** p<0.01, *** p<0.001, **** p<0.0001.

It has been reported that *Eomes* expression could elevated in the late stage of EAE 17. Our in-house study did replicate this finding (Figure 4A). To further validate the assumption that the increased *Eomes* expression was created by GK treatment, we tested the expression of *Eomes* at different stages of EAE (day 9 p.i., day 18 p.i., and day 28 p.i.). Despite that the total expression of *Eomes* was relatively lower when compared to which at day 28 p.i., the *Eomes* expression was significantly elevated in CD4^+^T cells after GK treatment at day 9 p.i. (Figure 4A). Moreover, we detected phospho JNK level in CD4^+^T cells (Figure 4B). Not surprisingly, the phospho JNK level was elevated in CD4^+^T cells at EAE day 18 compared to day 9, while GK treatment inhibited its elevation significantly (Figure 4B). As well, we found in GK treatment group, the level of phospho c-Jun, together with total c-Jun, was obviously lower than that in EAE group (Figure 4C). Meanwhile, the mRNA expression of Jun was showing the same trend (Figure 4D).

Since Eomes was also expressed on human CD4^+^T cells, we wondered if the promoter zone of human *EOMES* (*hEOMES*) was influenced when GK treatment either. Thus, we constructed Luciferase reporter gene plasmids containing different length of the* hEOMES* promoter (Figure 4E). Unsurprisingly, as shown in Figure 4E, the transcriptional activity differed among promoter sets containing different length of *hEOMES* promoter. After GK treatment (50μg/ml) for 24 hours, the transcriptional activity of promoter set 2, 3 and 4 increased slightly but significantly (Figure 4E). It had been well-demonstrated that one of the main downstream of phospho JNK, c-Jun, could directly bind to the promotor zone of *Eomes* and inhibit *Eomes* gene transcription 10. To replicated this result, we constructed pFLAG-c-Jun plasmid and transfected HEK293T cells along with the above Luciferase reporter gene plasmids. The relative activity of the *hEOMES* promoter was significantly decreased by nearly 50% (Figure 4F). These findings together demonstrated that GK increased the expression of *Eomes* indirectly through inhibiting the level of phospho JNK.

### Conditional ablation of *Eomes* in CD4^+^T cells restricted the effect of GK in EAE

**Figure 5:**
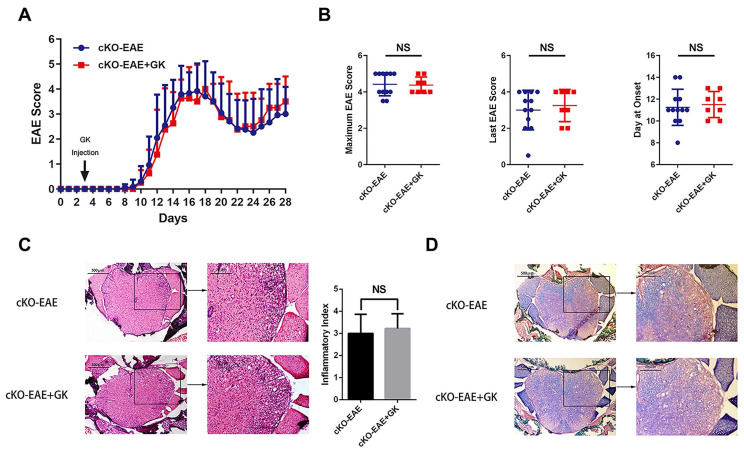
** Genetically ablation of *Eomes* in CD4^+^T cells interrupted the effects of GK treatment in EAE.** CD4-*Eomes*^cKO^ mice were immunized with MOG_35-55_ and injected intraperitoneally with 15mg per kilogram bodyweight GK once per day from day 3 p.i. to day 26 p.i. (A) EAE clinical score of GK treatment group (n=8) and vehicle control group (n=12). (B) The maximum EAE score, last EAE score and average day at onset of GK treatment group and vehicle control group. (C) H&E staining of GK treatment group and vehicle control group. (D) Luxol fast blue plus eosin staining of GK treatment group and vehicle control group. Data are representative of at least three independent experiments. Mean±SD is shown. NS Not significant, * p<0.05, ** p<0.01, *** p<0.001.

**Figure 6:**
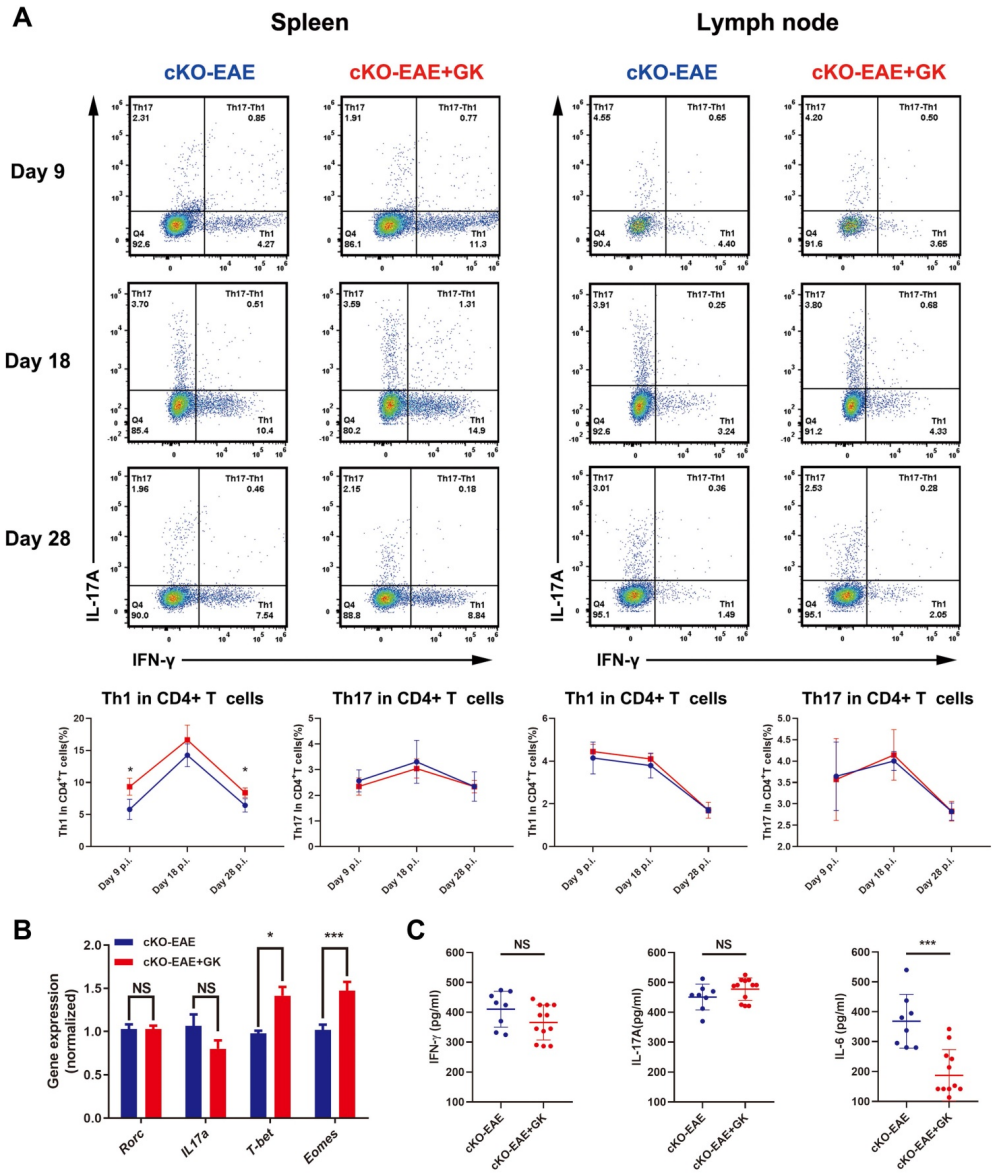
** GK treatment didn't decreased the level of inflammation in CD4-*Eomes*^cKO^ EAE.** (A) Intracellular cytokines IL-17A and IFN-γ staining by FACS at day 9 p.i., day 18 p.i. and day 28 p.i. (B) Spleen MNCs gene expression of* Rorc*, *Il17a*, *T-bet* and *Eomes* by qRT-PCR. (C) IFN-γ, IL-17A and IL-6 production in the serum of GK treatment group and vehicle control group. Data are representative of three independent experiments. Mean±SD is shown. NS Not significant, * p<0.05, ** p<0.01, *** p<0.001.

**Figure 7:**
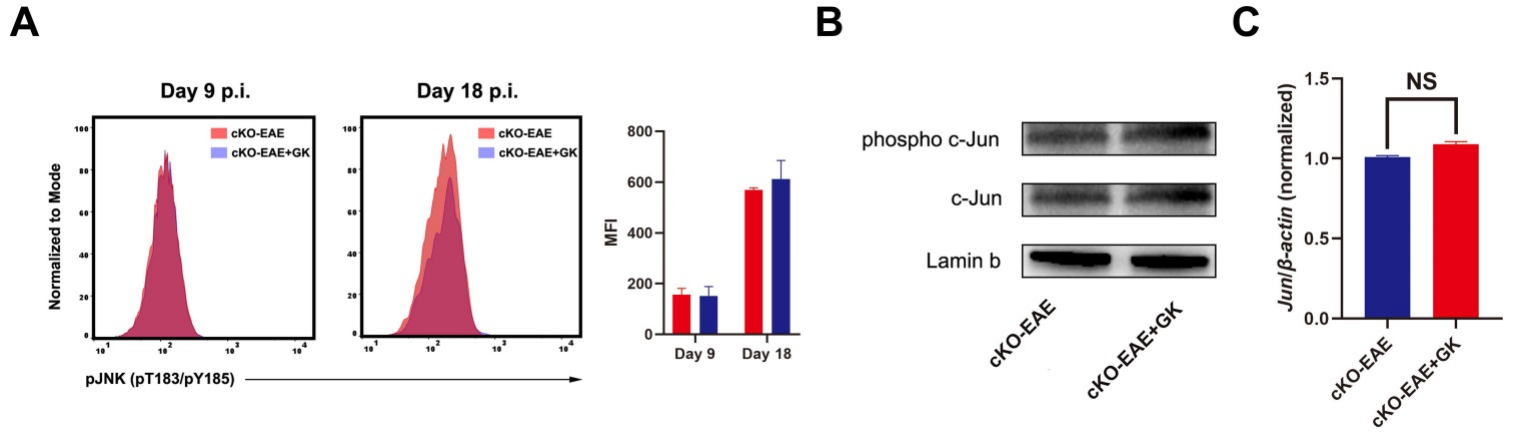
** The effect of GK treatment in CD4-*Eomes*^cKO^ EAE.** (A) The phospho JNK in spleen CD4^+^T cells at CD4-*Eomes*^cKO^ EAE day 9 p.i.and day 18 p.i. (B) The western blot of c-Jun and phospho c-Jun at CD4-*Eomes*^cKO^ EAE (day 18 p.i.) with or without GK treatment. (C) Spleen MNCs *Jun* expression between CD4-*Eomes*^cKO^ EAE with or without GK treatment. Data are representative of three independent experiments. Mean±SD is shown. NS Not significant, * p<0.05, ** p<0.01, *** p<0.001.

We have demonstrated that the expression of *Eomes* was elevated by GK treatment in EAE through inhibiting the level of phospho JNK, we wondered if elevated Eomes level is necessary for the effect of GK in the development of EAE. Thus, we performed EAE induction in CD4-* Eomes* conditional knockout mice (CD4-*Eomes*^cKO^). Interestingly, the clinical severity of EAE was not reduced in CD4-*Eomes*^cKO^ mice with GK treatment (Figure 5A). The maximum EAE score, the last EAE score and the average day at disease onset remained the same as CD4-*Eomes*^cKO^ mice treated with vehicle (Figure 5B). Histopathological evaluation revealed that both groups developed severe inflammation (Figure 5C) and demyelination (Figure 5D) in the spinal cords, in line with the clinical evaluation. Moreover, the intracellular cytokine staining showed no differences in the numbers of Th1 and Th17 cells among CD4^+^T cells both in spleen and lymph node (Figure 6A). Besides, we found a large amount of Eomes was deleted from CD8^+^T cells either in our CD4-*Eomes*^cKO^ mice. However, its portion in CD8^+^T cells was still increased after GK treatment (Supplemental Figure 1). QRT-PCR results indicated that the expression of *Rorc* was nearly the same after GK treatment, while the *Il17a* expression seemed decreased but without significance (Figure 6B). The total expression of *Eomes* in splenocytes still increased despite that it had been deleted in CD4^+^T cells (Figure 6B). Not surprisingly, the expression of *T-bet* was slightly increased after GK treatment, considering it might compensate the loss of Eomes 18. The IL-6 secretion in the serum of CD4-*Eomes*^cKO^ EAE mice with GK treatment was still much lower than which without GK. Nevertheless, the IFN-γ and IL-17A remained not changed (Figure 6C). The phospho JNK was remained unchanged in CD4^+^T cells, as well as the c-Jun and phospho c-Jun (Figure 7A-C). After all, these results in CD4-*Eomes*^cKO^ EAE mice demonstrated the importance and necessity of CD4^+^ Eomes^+^ T cells in GK treatment.

## Discussion

MS is considered as one of the most common causes of neurological disability in young adults 1. Th17 cell disbalance was widely believed to be crucially involved in the pathogenesis of MS, as well as EAE model. EAE model has been proven to be helpful in testing new concepts or therapies 13, 19. Here, we found a low MW compound, Ginkgolide K, can ameliorate the disease progression of EAE by regulating the development of Th17 cells. We further demonstrated that the expression of *Eomes* increased after GK treatment, while Eomes in CD4^+^T cells is essential for the effect of GK treatment.

In Th cell differentiation, transcription factors are playing crucial roles. For instance, T-bet, Foxp3 and RORγt are the master transcription factors of the Th1, Treg and Th17 cells, respectively. However, the role of Eomes, especially in CD4^+^T cells, remains controversial. On one hand, the expression of *Eomes* may increase the production of IFN-γ 20, as well as GM-CSF, perforin and granzyme B 21, 22. On the other hand, Eomes itself can bind to the promotor zone of *Rorc* and *Il17a* and limit the development of Th17 cells via a Smad-independent pathway 10. One study demonstrated that the expression of *Eomes* restricts the peripheral Foxp3 Induction, especially in elder mice 23. However, Zhang and colleagues reported that Eomes promotes the development of type 1 regulatory T (Tr1) cells, a *Foxp3* negative regulatory T cell subset, which might have potent immunosuppressive functions in autoimmunity 24. Even more recently, it has been further demonstrated that Eomes controls the development of Th17-derived (non-classic) Th1 cells and repress *Rorc2* and *Il17a* in Th17 cells 25. Thus, the role Eomes played in autoimmunity depends more on which cell type it expresses in and what intervention we gave. In the present study, we found that accompanied by increased expression of *Eomes*, GK treatment significantly ameliorated the EAE disease progression.

It has been proposed that *Eomes* drives the development of pathogenic Th1 cells in EAE by favoring the contemporary secretion of IFN-γ and GMCSF, that is needed for the development of central nervous system inflammation 22. Besides, in line with a previous report 17, we also found that *Eomes* expression could elevated in the late stage of wildtype EAE. Thus, Eomes may favor the phenotype shift of Th17 cells towards Th1 by reinforcing IFN-γ production and induce a late stage inflammation. Contrary to this result, we found that GK-induced Eomes didn't drive the development of pathogenic Th1 cells and production of IFN-γ in both WT and CD4-*Eomes*^cKO^ mice. We assumed it might be due to the anti-inflammation ability of GK as a powerful platelet-activating factor (PAF) receptor antagonist 26. As was reported, PAFR blockade in EAE can attenuate the disease progression and lower the CNS inflammatory infiltrations 27, especially the IFN-γ producing in the early stage of EAE 28. Additionally, the IL-6 were reported significantly reduced by 43% by PAF receptor blockage 26. IL-6 is an essential factor for Th17 cell differentiation. Eomes induction, together with IL-6 and IFN-γ blockade, inhibited the differentiation of Th17 and Th1 cells and, thus, the development of EAE 29-31. However, it still remains to be elucidated the role of Eomes^+^ CD4^+^T cells and Eomes^high^ CD4^+^T cells in the development of EAE.

**Figure 8:**
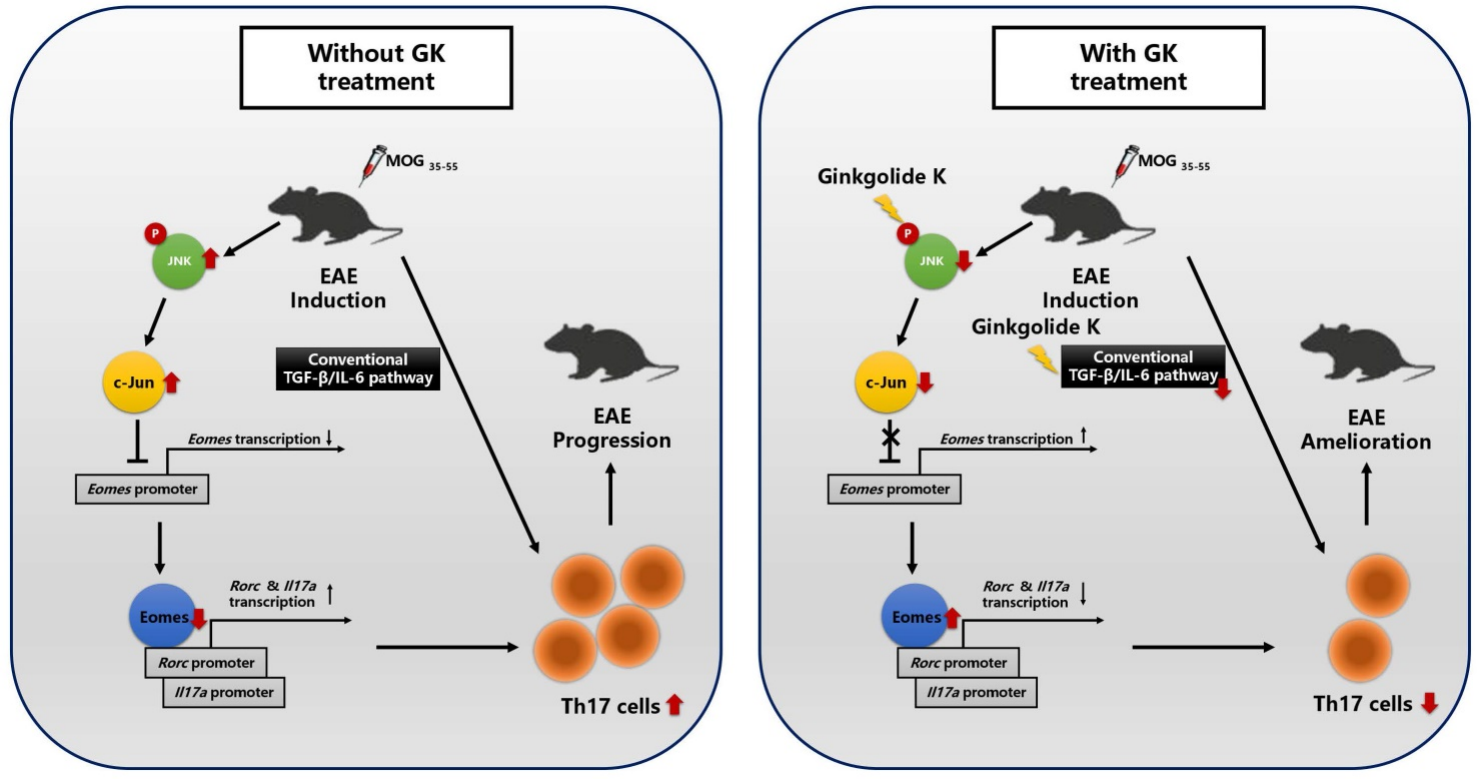
The discrepancy in molecular process between EAEs with or without GK treatment.

How to understand that GK did not alleviate EAE disease progression in CD4-*Eomes*^cKO^ mice? It has been reported that suppression of Eomes via TGF-β-JNK-c-Jun pathway can promote full Th17 induction 10. Moreover, GK may interrupt the interaction between phospho IRE1α and Traf2, and restrict the activation of JNK in NRMCs 12. In the present study, we found that GK also decreased the level of phospho JNK and c-Jun in lymphocyte, and thus increased the expression of *Eomes*. This effect presented not only in CD4^+^T cells, but also in CD4^-^T cells and throughout the whole process of EAE while GK treatment. On the other hand, the expression of *Eomes* in CD4^+^T cells is essential for the effect of GK, because the conditional ablation of *Eomes* in CD4^+^T cells almost completely interrupted the effect of GK in EAE model. It reminded us that to decrease the phospho JNK and increase the level of Eomes might be the major target pathway of GK treatment in EAE model (Figure 8). We assumed it was because that the Th17 induction in wildtype EAE mice was following both the traditional TGF-β-IL-6 pathway and the SMAD-independent TGF-β-JNK-c-Jun pathway. GK treatment targeted to IL-6 and blocked the traditional TGF-β-IL-6 pathway. Meanwhile, Eomes induction block the SMAD-independent TGF-β-JNK-c-Jun pathway. Thus, the Th17 development in wildtype EAE mice was significantly attenuated. Nevertheless, GK treatment blocked IL-6 either in Eomes knockout EAE mice, but failed to block the TGF-β-JNK-c-Jun pathway. In this situation, Th17 inflammation could be fully induced.

Little is known whether it is useful for GK treatment in MS patients, however there does exist several clinical evidences between decreased Eomes level and the occurrence of MS. Another study had demonstrated that the mRNA expression of several transcription factors, including *EOMES*, was significantly and consistently lower in MS patients comparing to healthy controls 32. Moreover, a number of genome-wide association studies (GWAS) together with our previous study had recognized *EOMES* gene polymorphisms as risk loci 33-35. Interestingly, the reported risk loci were all located in the noncoding zone of *EOMES*, which might be associated with the regulation of *EOMES* expression.

In summary, our results demonstrated that GK has a robust therapeutic effect on the disease progression in EAE. This therapeutic effect is based on the elevation of Eomes in CD4^+^T cells and the inhibition of phospho JNK, revealing that Eomes in CD4^+^T cells is essential for the effect of GK treatment. Our findings may suggest a new insight of Eomes in autoimmunity and provide a novel potential therapeutic approach for MS.

## Supplementary Material

Supplementary figure and table.

## Abbreviations

CFA: complete Freund's adjuvant
CNS: central nervous system
EAE: experimental autoimmune encephalomyelitis
GK: Ginkgolide K
GWAS: genome-wide association study
JNK: c-Jun N-terminal kinase
KO: knockout
MNC: Mononuclear cell
MS: multiple sclerosis
MW: molecular weight
NRCM: neonatal rat cardiomyocyte
PAF: platelet-activating factor
PCR: polymerase chain reaction
TF: transcription factor

## References

[1] Compston A, Coles A (2008). Multiple sclerosis. Lancet.

[2] Constantinescu CS, Farooqi N, O'Brien K, Gran B (2011). Experimental autoimmune encephalomyelitis (EAE) as a model for multiple sclerosis (MS). Br J Pharmacol.

[3] Szabo SJ, Kim ST, Costa GL, Zhang X, Fathman CG, Glimcher LH (2000). A novel transcription factor, T-bet, directs Th1 lineage commitment. Cell.

[4] Fontenot JD, Gavin MA, Rudensky AY (2003). Foxp3 programs the development and function of CD4+CD25+ regulatory T cells. Nat Immunol.

[5] Hori S, Nomura T, Sakaguchi S (2003). Control of regulatory T cell development by the transcription factor Foxp3. Science.

[6] Harrington LE, Hatton RD, Mangan PR, Turner H, Murphy TL, Murphy KM (2005). Interleukin 17-producing CD4+ effector T cells develop via a lineage distinct from the T helper type 1 and 2 lineages. Nat Immunol.

[7] Korn T, Bettelli E, Gao W, Awasthi A, Jager A, Strom TB (2007). IL-21 initiates an alternative pathway to induce proinflammatory T(H)17 cells. Nature.

[8] Ivanov II, McKenzie BS Zhou L, Tadokoro CE Lepelley A, Lafaille JJ et al (2006). The orphan nuclear receptor RORgammat directs the differentiation program of proinflammatory IL-17+ T helper cells. Cell.

[9] Yang XO, Pappu BP, Nurieva R, Akimzhanov A, Kang HS, Chung Y (2008). T helper 17 lineage differentiation is programmed by orphan nuclear receptors ROR alpha and ROR gamma. Immunity.

[10] Ichiyama K, Sekiya T, Inoue N, Tamiya T, Kashiwagi I, Kimura A (2011). Transcription factor Smad-independent T helper 17 cell induction by transforming-growth factor-beta is mediated by suppression of eomesodermin. Immunity.

[11] Yu WB, Wang Q, Chen S, Cao L, Tang J, Ma CG (2019). The therapeutic potential of ginkgolide K in experimental autoimmune encephalomyelitis via peripheral immunomodulation. Int Immunopharmacol.

[12] Wang S, Wang Z, Fan Q, Guo J, Galli G, Du G (2016). Ginkgolide K protects the heart against endoplasmic reticulum stress injury by activating the inositol-requiring enzyme 1alpha/X box-binding protein-1 pathway. Br J Pharmacol.

[13] Su P, Chen S, Zheng YH, Zhou HY, Yan CH, Yu F (2016). Novel Function of Extracellular Matrix Protein 1 in Suppressing Th17 Cell Development in Experimental Autoimmune Encephalomyelitis. J Immunol.

[14] Muramatsu R, Kubo T, Mori M, Nakamura Y, Fujita Y, Akutsu T (2011). RGMa modulates T cell responses and is involved in autoimmune encephalomyelitis. Nat Med.

[15] von Palffy S, Landberg N, Sanden C, Zacharaki D, Shah M, Nakamichi N (2020). A high-content cytokine screen identifies myostatin propeptide as a positive regulator of primitive chronic myeloid leukemia cells. Haematologica.

[16] Hsin KY, Matsuoka Y, Asai Y, Kamiyoshi K, Watanabe T, Kawaoka Y (2016). systemsDock: a web server for network pharmacology-based prediction and analysis. Nucleic Acids Res.

[17] Raveney BJ, Oki S, Hohjoh H, Nakamura M, Sato W, Murata M (2015). Eomesodermin-expressing T-helper cells are essential for chronic neuroinflammation. Nat Commun.

[18] Yi CH, Terrett JA, Li QY, Ellington K, Packham EA, Armstrong-Buisseret L (1999). Identification, mapping, and phylogenomic analysis of four new human members of the T-box gene family: EOMES, TBX6, TBX18, and TBX19. Genomics.

[19] Liu CY, Guo SD, Yu JZ, Li YH, Zhang H, Feng L (2015). Fasudil mediates cell therapy of EAE by immunomodulating encephalomyelitic T cells and macrophages. Eur J Immunol.

[20] Suto A, Wurster AL, Reiner SL, Grusby MJ (2006). IL-21 inhibits IFN-gamma production in developing Th1 cells through the repression of Eomesodermin expression. J Immunol.

[21] Pearce EL, Mullen AC, Martins GA, Krawczyk CM, Hutchins AS, Zediak VP (2003). Control of effector CD8+ T cell function by the transcription factor Eomesodermin. Science.

[22] Stienne C, Michieletto MF, Benamar M, Carrie N, Bernard I, Nguyen XH (2016). Foxo3 Transcription Factor Drives Pathogenic T Helper 1 Differentiation by Inducing the Expression of Eomes. Immunity.

[23] Lupar E, Brack M, Garnier L, Laffont S, Rauch KS, Schachtrup K (2015). Eomesodermin Expression in CD4+ T Cells Restricts Peripheral Foxp3 Induction. J Immunol.

[24] Zhang P, Lee JS, Gartlan KH, Schuster IS, Comerford I, Varelias A (2017). Eomesodermin promotes the development of type 1 regulatory T (TR1) cells. Sci Immunol.

[25] Mazzoni A, Maggi L, Siracusa F, Ramazzotti M, Rossi MC, Santarlasci V (2018). Eomes controls the development of Th17-derived (non-classic) Th1 cells during chronic inflammation. Eur J Immunol.

[26] Zhang H, Yang Y, Takeda A, Yoshimura T, Oshima Y, Sonoda KH (2013). A Novel Platelet-Activating Factor Receptor Antagonist Inhibits Choroidal Neovascularization and Subretinal Fibrosis. PLoS One.

[27] Rodrigues DH, Lacerda-Queiroz N, de Miranda AS, Fagundes CT, Campos RD, Arantes RE (2011). Absence of PAF receptor alters cellular infiltrate but not rolling and adhesion of leukocytes in experimental autoimmune encephalomyelitis. Brain Res.

[28] Kihara Y, Ishii S, Kita Y, Toda A, Shimada A, Shimizu T (2005). Dual phase regulation of experimental allergic encephalomyelitis by platelet-activating factor. J Exp Med.

[29] Serada S, Fujimoto M, Mihara M, Koike N, Ohsugi Y, Nomura S (2008). IL-6 blockade inhibits the induction of myelin antigen-specific Th17 cells and Th1 cells in experimental autoimmune encephalomyelitis. Proc Natl Acad Sci U S A.

[30] Korn T, Mitsdoerffer M, Croxford AL, Awasthi A, Dardalhon VA, Galileos G (2008). IL-6 controls Th17 immunity in vivo by inhibiting the conversion of conventional T cells into Foxp3+ regulatory T cells. Proc Natl Acad Sci U S A.

[31] Yang C, Lai W, Zhou J, Zheng X, Cai Y, Yang W (2018). Betaine Ameliorates Experimental Autoimmune Encephalomyelitis by Inhibiting Dendritic Cell-Derived IL-6 Production and Th17 Differentiation. J Immunol.

[32] Parnell GP, Gatt PN, Krupa M, Nickles D, McKay FC, Schibeci SD (2014). The autoimmune disease-associated transcription factors EOMES and TBX21 are dysregulated in multiple sclerosis and define a molecular subtype of disease. Clin Immunol.

[33] International Multiple Sclerosis Genetics C, Wellcome Trust Case Control C, Sawcer S, Hellenthal G, Pirinen M, Spencer CC (2011). Genetic risk and a primary role for cell-mediated immune mechanisms in multiple sclerosis. Nature.

[34] International Multiple Sclerosis Genetics C, Beecham AH, Patsopoulos NA, Xifara DK, Davis MF, Kemppinen A (2013). Analysis of immune-related loci identifies 48 new susceptibility variants for multiple sclerosis. Nat Genet.

[35] Chen S, Zhang J, Liu QB, Zhuang JC, Wu L, Xu YF (2018). Variant of EOMES Associated with Increasing Risk in Chinese Patients with Relapsing-remitting Multiple Sclerosis. Chin Med J (Engl).

