# Supplementary material for: Eomesodermin in CD4+T cells is essential for Ginkgolide K ameliorating disease progression in experimental autoimmune encephalomyelitis
Source: Int J Biol Sci. 2021 Jan 1;17(1):50–61. doi: 10.7150/ijbs.50041 (PMC7757039; doi:10.7150/ijbs.50041)

Supplementary table 1. Primer pairs for qRT-PCR

Name	Species	Sequence
mRORC-q-F	Mus musculus	AGCTGCGACTGGAGGACCTT
mRORC-q-R	Mus musculus	CCCGTGAAAAGAGGTTGGTG
mEOMES-q-F	Mus musculus	GTGACGGCCTACCAAAACAC
mEOMES-q-R	Mus musculus	GACCTCCAGGGACAATCTGA
mT-bet-q-F	Mus musculus	CCATTCCTGTCCTTCACCGT
mT-bet-q-R	Mus musculus	CCTGTAATGGCTTGTGGGCT
mFOXP3-q-F	Mus musculus	CACCTATGCCACCCTTATCC
mFOXP3-q-R	Mus musculus	GAAGTAGGCGAACATGCGAG
mIL17a-F	Mus musculus	CACCTCACACGAGGCACAAG
mIL17a-R	Mus musculus	CAGCAACAGCATCAGAGACACA
mJun-F	Mus musculus	TGGGCACATCACCACTACAC
mJun-R	Mus musculus	GACACTGGGAAGCGTGTTCT
mβ-actin-F	Mus musculus	GAGACCTTCAACACCCCAG
mβ-actin-R	Mus musculus	CATCACAATGCCTGTGGTAC

Supplemental figure 1

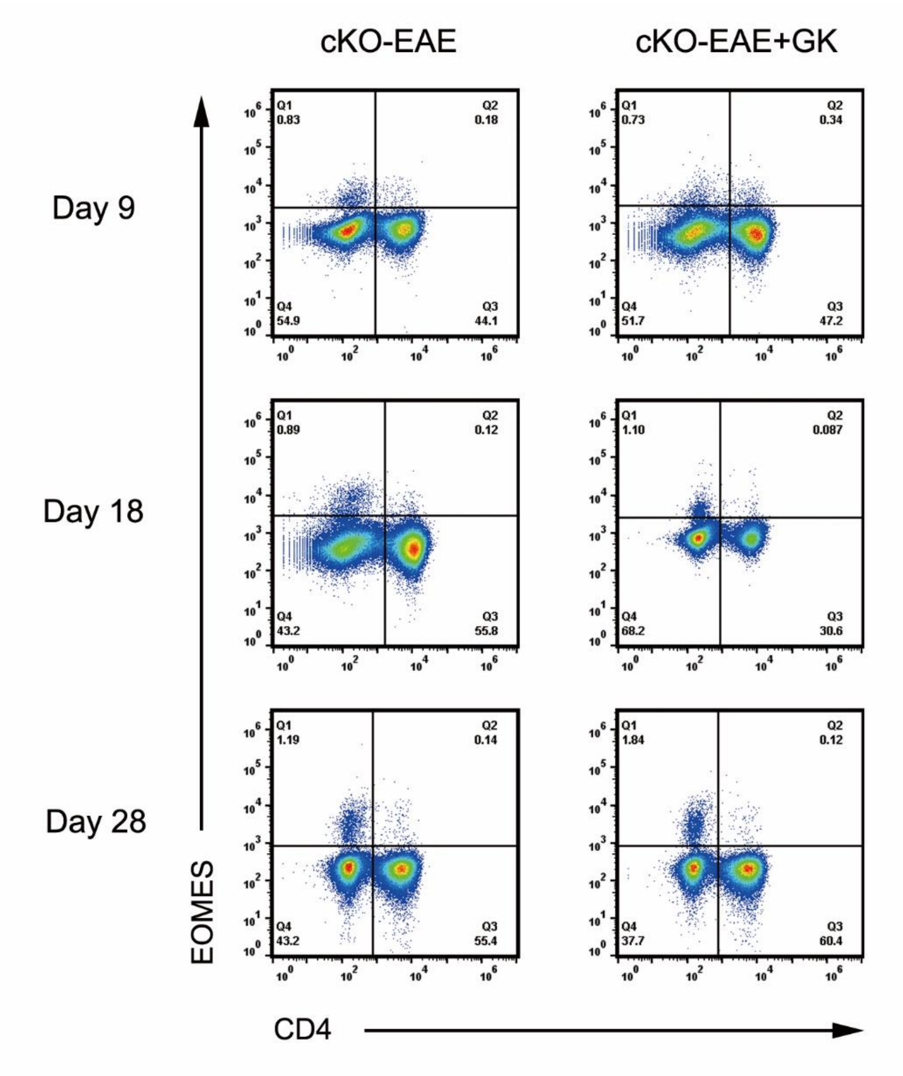

Supplement: Supplementary file 1 — Supplementary figure and table. [file ijbsv17p0050s1.pdf]
